# MORTAR: a rich client application for in silico molecule fragmentation

**DOI:** 10.1186/s13321-022-00674-9

**Published:** 2023-01-02

**Authors:** Felix Bänsch, Jonas Schaub, Betül Sevindik, Samuel Behr, Julian Zander, Christoph Steinbeck, Achim Zielesny

**Affiliations:** 1 Institute for Bioinformatics and Chemoinformatics, Westphalian University of Applied Sciences, August-Schmidt-Ring 10, 45665 Recklinghausen, Germany; 2 Institute for Inorganic and Analytical Chemistry, Friedrich Schiller University Jena, Lessingstraße 8, 07743 Jena, Germany

**Keywords:** Chemistry Development Kit, CDK, Molecule fragmentation, In silico fragmentation, Scaffolds, Functional groups, Glycosidic moieties, Rich client, Graphical user interface, GUI

## Abstract

Developing and implementing computational algorithms for the extraction of specific substructures from molecular graphs (in silico molecule fragmentation) is an iterative process. It involves repeated sequences of implementing a rule set, applying it to relevant structural data, checking the results, and adjusting the rules. This requires a computational workflow with data import, fragmentation algorithm integration, and result visualisation. The described workflow is normally unavailable for a new algorithm and must be set up individually. This work presents an open Java rich client Graphical User Interface (GUI) application to support the development of new in silico molecule fragmentation algorithms and make them readily available upon release. The MORTAR (MOlecule fRagmenTAtion fRamework) application visualises fragmentation results of a set of molecules in various ways and provides basic analysis features. Fragmentation algorithms can be integrated and developed within MORTAR by using a specific wrapper class. In addition, fragmentation pipelines with any combination of the available fragmentation methods can be executed. Upon release, three fragmentation algorithms are already integrated: ErtlFunctionalGroupsFinder, Sugar Removal Utility, and Scaffold Generator. These algorithms, as well as all cheminformatics functionalities in MORTAR, are implemented based on the Chemistry Development Kit (CDK).

**Graphical Abstract:**

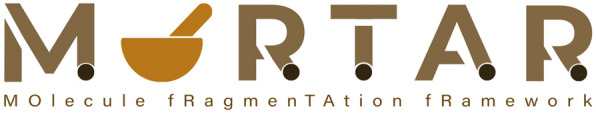

## Introduction

In quantum mechanics, molecular systems are holistic entities whose properties can only be fully understood by studying their complete structures. However, a reductionist approach that considers a molecular structure as a sum of multiple substructures has proven useful. A prominent example is the concept of functional groups (FG), which allows an assessment of molecular reactivity or toxicity based on specific FG occurrence. The concept is important for nomenclature generation, spectroscopy, combinatorial chemistry, or drug development as well [1].

The study of molecules based on their substructures is also widely applicable in cheminformatics. For many applications, lists of specific substructural patterns are compiled and matched against input structures, e.g., for indexing of structural databases to allow faster substructure searching [2–4], for the automatic detection of functional groups [5, 6], or for molecular fingerprints based on pre-defined structural keys [3, 7, 8]. Circular or spherical substructures are extracted from input molecules, i.e., fragments that each represent one atom and its neighbours up to a certain degree, to generate circular or path-based fingerprints [8–11], HOSE codes for computer-assisted structure elucidation (CASE) [12], and molecular signatures [13–16]. Circular substructures can be used to assess and compare chemical diversity in a given data set or between multiple sets. For example, estimations can be made about the natural product-likeness of a compound by comparing its circular substructures to a curated data set of those frequently appearing in secondary metabolites or synthetic molecules [17–20]. Another substructure concept frequently applied for chemical diversity assessment is the grouping of compounds by their molecular framework or scaffold. It is also used intuitively in combinatorial chemistry or drug design. Structural scaffold definitions in cheminformatics can be found in Murcko frameworks [21], scaffold trees [22–24], or scaffold networks [25, 26], which are all based on ring systems and the linear substructures connecting them. For scaffold trees, Schuffenhauer et al. defined chemical rules to dissect molecular scaffolds into their characteristic smaller parent scaffolds that allow for hierarchical classification of compounds when placed in the scaffold tree [22]. Such chemical expert rule systems can be grouped under the term “molecule fragmentation”, i.e., algorithmic extraction of specific substructures from input molecules in silico. Another example is the Ertl algorithm for automated in silico FG detection in organic molecules [27], which overcomes the need for manually-curated lists of FG substructure patterns. With this approach, the complete diversity of functional groups can be studied and incorporated into chemical space mappings and molecular fragment descriptors [28]. A similar algorithmic method was developed for the identification and removal of glycosidic moieties from organic molecules [29]. The Sugar Removal Utility (SRU) can be used for deglycosylation as a preprocessing step in chemical space analyses and was also employed to document the diversity of sugar moieties in the largest open natural product (NP) database COCONUT [30, 31].

Fragmentation algorithms like the ones mentioned above can be seen as distillates from chemical expert knowledge that allow computer programs to identify characteristic molecular substructures in a comprehensible way based on structural definitions for functional concepts like functional groups. Their advantage over methods employing pre-defined lists of substructure patterns is that they are complete, i.e., they capture the true diversity of substructures like functional groups, scaffolds, or glycosidic moieties. These traits make them important for fields like drug design, CASE, or NP research.

The development of fragmentation algorithms is often an iterative process with multiple steps of defining a rule set, applying it to suitable structural data, inspecting the results, and refining the algorithm based on what was observed in the previous step. This requires a testing workflow with fragment visualisation functions where two perspectives are of interest: One based on an individual molecule, i.e., which fragments result from its structure. The other considers a complete data set, asking which fragments occur how often in a set of structures. When a new fragmentation algorithm is ready for publication, it is usually released to the scientific community as a stand-alone command-line tool or as part of a cheminformatics programming library. If users want to visually inspect the fragment set generated by the new algorithm for their own set of molecules, they have to implement corresponding workflows themselves.

This work presents an open Java rich client Graphical User Interface (GUI) application, abbreviated MORTAR (MOlecule fRagmenTAtion fRamework), with the intention to support the development of new in silico molecule fragmentation algorithms as well as enable their later distribution in a more accessible way. Its main functionalities are importing molecule sets from various file formats, applying fragmentation algorithms, and visualising the results in a graphical display that alleviates the investigation of the resulting fragment sets and the fragmentation results of individual molecules. Using MORTAR, no programming skills are needed to perform these steps, which makes conducting in silico fragmentation studies more accessible. MORTAR can be installed and used on three major operating systems (Windows, macOS, and Linux) and uses parallel computing on multi-core processors for CPU-intensive processes. To our knowledge, there is currently no comparable open software application available. For cheminformatics functionalities, the Chemistry Development Kit (CDK) [32–34] is employed internally. Fragmentation algorithms can be integrated into or directly developed in MORTAR straightforwardly. Upon release, three algorithms are available: The Ertl algorithm for functional group identification via its open implementation ErtlFunctionalGroupsFinder [35], the Sugar Removal Utility for glycosidic moiety detection and removal, and Scaffold Generator [36], a software library for scaffold functionalities, including the scaffold tree dissection into parent scaffolds and an enumerative parent scaffold generation routine for scaffold networks. More functionalities will be added in future releases and with MORTAR being open-source, the software itself can be enhanced or tailored to individual needs.

## Implementation

MORTAR was implemented as a rich client application based on Java 17 using Adoptium OpenJDK [37]. Gradle [38] was used to facilitate the build process and the integration of further libraries. All libraries used are obtained from the Maven Central Repository [39].

The architecture of MORTAR follows a Model-View-Controller (MVC) pattern based on the object-oriented Java framework. MORTAR is available on GitHub as a free open-source project: https://github.com/FelixBaensch/MORTAR. The repository contains the complete source code, an extensive graphical tutorial explaining every detail of the GUI, and an installation guide.

The class structure is organised according to the MVC pattern with one package for each layer. The view classes are designed with JavaFX [40] and located in their respective sub-packages of the *gui* package: *controls*, *util*, and *views*. These classes are governed by a layer of controller classes which are located in the *controller* package. The controllers pass on the user input to a layer of model classes and update the GUI according to the resulting changes in the data model.

Two data model classes represent the molecules and their fragments: *MoleculeDataModel* and *FragmentDataModel*. For all chemical functionalities and chemical data processing, the Chemistry Development Kit (CDK) is used. Imported molecules are represented as objects of *MoleculeDataModel*, a class that holds several properties of the corresponding molecule. More memory-intensive properties, such as the 2D structure image, are only created when needed and then released afterwards for the Java garbage collector to discard. Similarly, the central CDK data structure for the representation of chemical molecules, *IAtomContainer*, is only created on demand from the SMILES [41] representation of a molecule or fragment. SMILES line notations are internally used as the only retained molecular structure information to save memory. This is useful when loading large molecule sets and allows working on less powerful computer systems. This way, MORTAR is able to import the complete COCONUT database [42] with more than 400,000 natural products on a standard laptop with only 16 GB RAM.

Fragments are represented as objects of *FragmentDataModel*, a class that extends *MoleculeDataModel*, to provide properties such as the frequency of the fragment or the number of molecules in which the specific fragment appears.

**Fig. 1:**
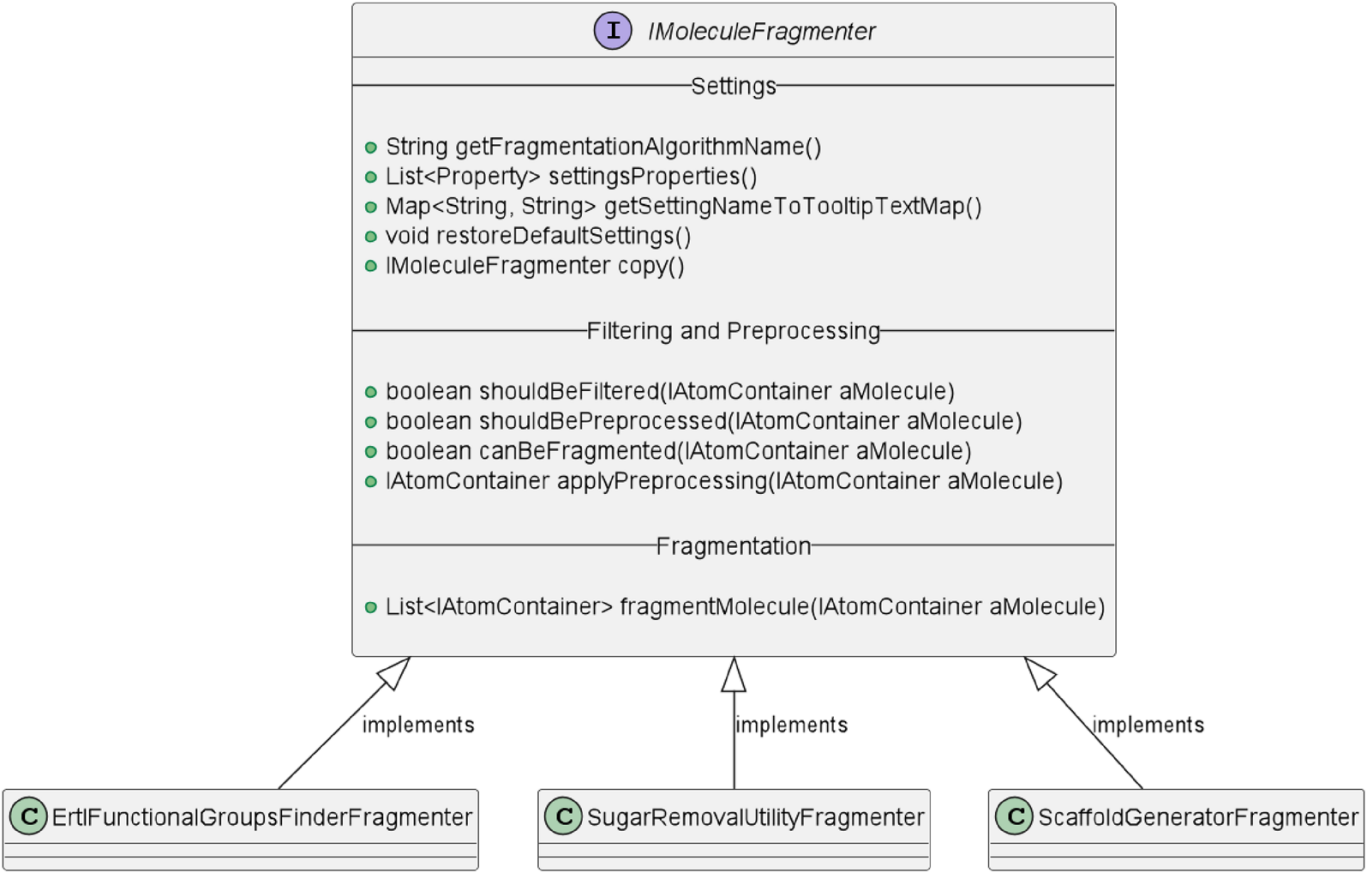
Class diagram of the *IMoleculeFragmenter* interface with its central methods and implementing classes, created using PlantUML [43, 44]

The controller layer manages the integrated fragmentation algorithms and sets up the fragmentation jobs. Currently, three methods of fragmentation and substructure analysis are integrated: ErtlFunctionalGroupsFinder, Sugar Removal Utility (SRU), and Scaffold Generator. Each of these methods is a stand-alone implementation based on CDK. Each fragmentation algorithm implementation has an individual wrapper class that implements a MORTAR-specific interface called *IMoleculeFragmenter*. Using this interface (see Fig. 1), fragmentation algorithms can be integrated into MORTAR.

**Fig. 2:**
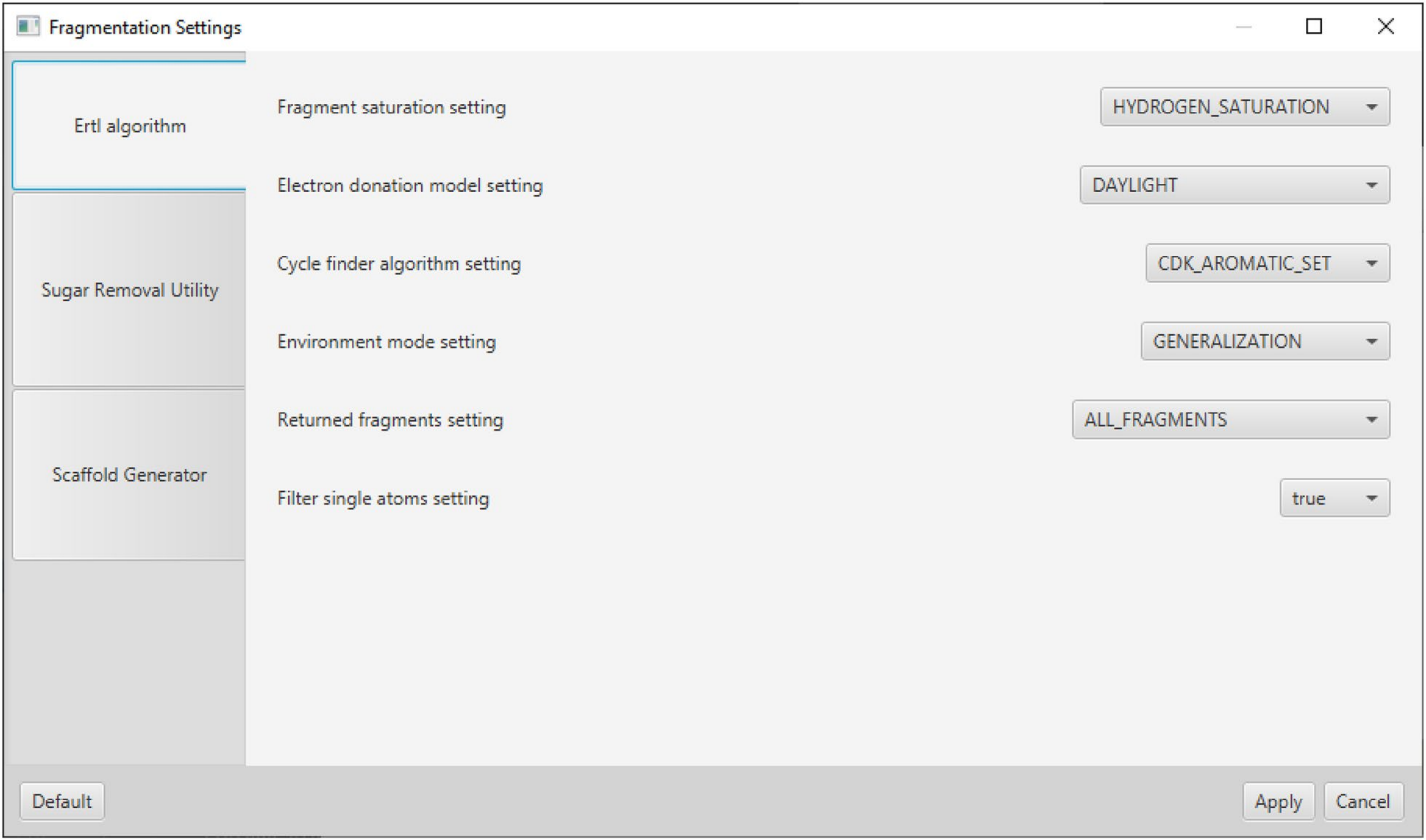
Settings view for the already integrated fragmentation algorithms. Settings for ErtlFunctionalGroupsFinder (Ertl algorithm) are selected

Specific fragmentation settings of an algorithm must be implemented as objects of type-specific property classes implementing the JavaFX *Property* < *T* > interface and returned in a list by the *IMoleculeFragmenter* method *settingsProperties()*. This way, a specific tab for the algorithm is automatically generated in the *SettingsView* of the GUI with text fields for strings or numbers or a choice box based on the property types (see Fig. 2). Tooltip texts describing the settings can also be defined. This auto-creation of a settings dialog supports the convenience of integrating new fragmentation algorithms into MORTAR. Apart from the settings, conditions for the input molecules passed to the specific algorithm or preprocessing routines can be defined via the interface. For example, the *ErtlFunctionalGroupsFinder* class does not accept input structures containing metal or metalloid atoms or charges. The former have to be filtered, the latter can be neutralised in preprocessing. The central fragmentation method in the interface is *fragmentMolecule()* which requires a molecule represented by an *IAtomContainer* object as input and returns a list of fragments represented by the same class. Here, the fragmentation algorithm logic must be implemented. Well-documented example code can be found in the three classes *ErtlFunctionalGroupsFinderFragmenter*, *SugarRemovalUtilityFragmenter*, and *ScaffoldGeneratorFragmenter*.

The MORTAR class *FragmentationService* keeps track of the available fragmentation algorithm classes, i.e., it persists and reloads their settings between sessions and manages the fragmentation process. The latter is started by passing a list of input molecules to the *FragmentationService* class. One by one, these are processed with the *IMoleculeFragmenter.fragmentMolecule()* method of the currently selected fragmentation algorithm, which returns a list of fragment structures for every input molecule. In post-processing, each molecule object receives a list containing the fragments and their frequencies in the molecule for the specific fragmentation run. If multiple fragmentations are performed, another fragmentation run-specific list is added. In addition to executing fragmentation steps individually, fragmentation pipelines with multiple steps can be defined and executed with any combination of the integrated fragmentation methods.

For high-performance processing, especially for larger molecule sets, MORTAR uses parallel computing with multiple threads. The set of input molecules is divided onto a number of parallel threads specified by the user, these subsets are fragmented in parallel, and the results are then recombined to create the coherent final set of fragments.

MORTAR logs internal information and problems automatically using the Java logging API. A log file is written for every application session and can be accessed by the user.

## Results and discussion

MORTAR aims to support workflows for molecular in silico fragmentation and substructure analysis as well as the development of new fragmentation methods. The Java rich client application provides a graphical user interface for visualising the fragmentation results of individual molecular compounds or whole sets of molecules. It supports performing fragmentation with a single fragmentation algorithm or with a pipeline that can be any combination of the integrated fragmentation algorithms. Furthermore, MORTAR allows straightforward integration of additional fragmentation and substructure analysis methods.

### Import and molecules tab

Single molecular compounds or sets of molecules can be imported into MORTAR from various file formats. Text files containing SMILES codes and structure-data files (SDF) [45] can be used to import molecule sets. Individual molecular compounds can be read as Molfiles, where V2000 and V3000 are supported [45].

**Fig. 3:**
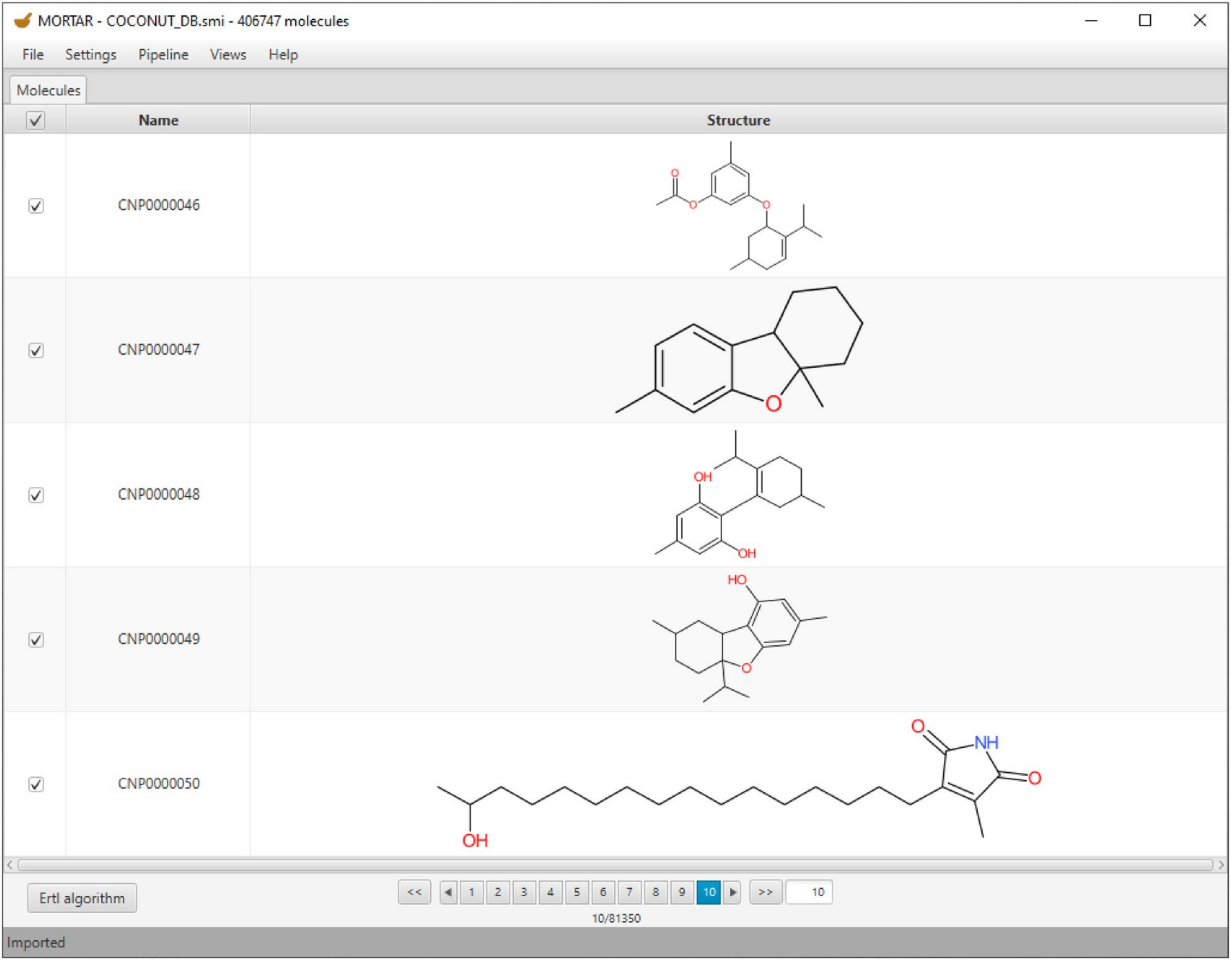
*Molecules* tab displaying imported molecules from the COCONUT database. Pagination allows browsing through the entire imported data set. The fragmentation job with the selected algorithm (in this case, the Ertl algorithm for functional group identification) can be started with the button at the bottom left

Figure 3 depicts the MORTAR *Molecules* tab displaying the structures of the COCONUT natural products database imported from a SMILES file. They can be browsed with a pagination tool and sorted by name in the table. Moreover, the molecules can be selected or deselected for fragmentation.

A single fragmentation process with a selected fragmentation algorithm can be started with the button in the bottom left corner. It is labelled with the selected fragmentation algorithm, which can be changed in a respective menu. The Ertl algorithm for functional group detection is selected for the fragmentation process in Fig. 3. In its MORTAR implementation, it returns identified functional groups and resulting alkane remnants as fragments by default.

**Fig. 4:**
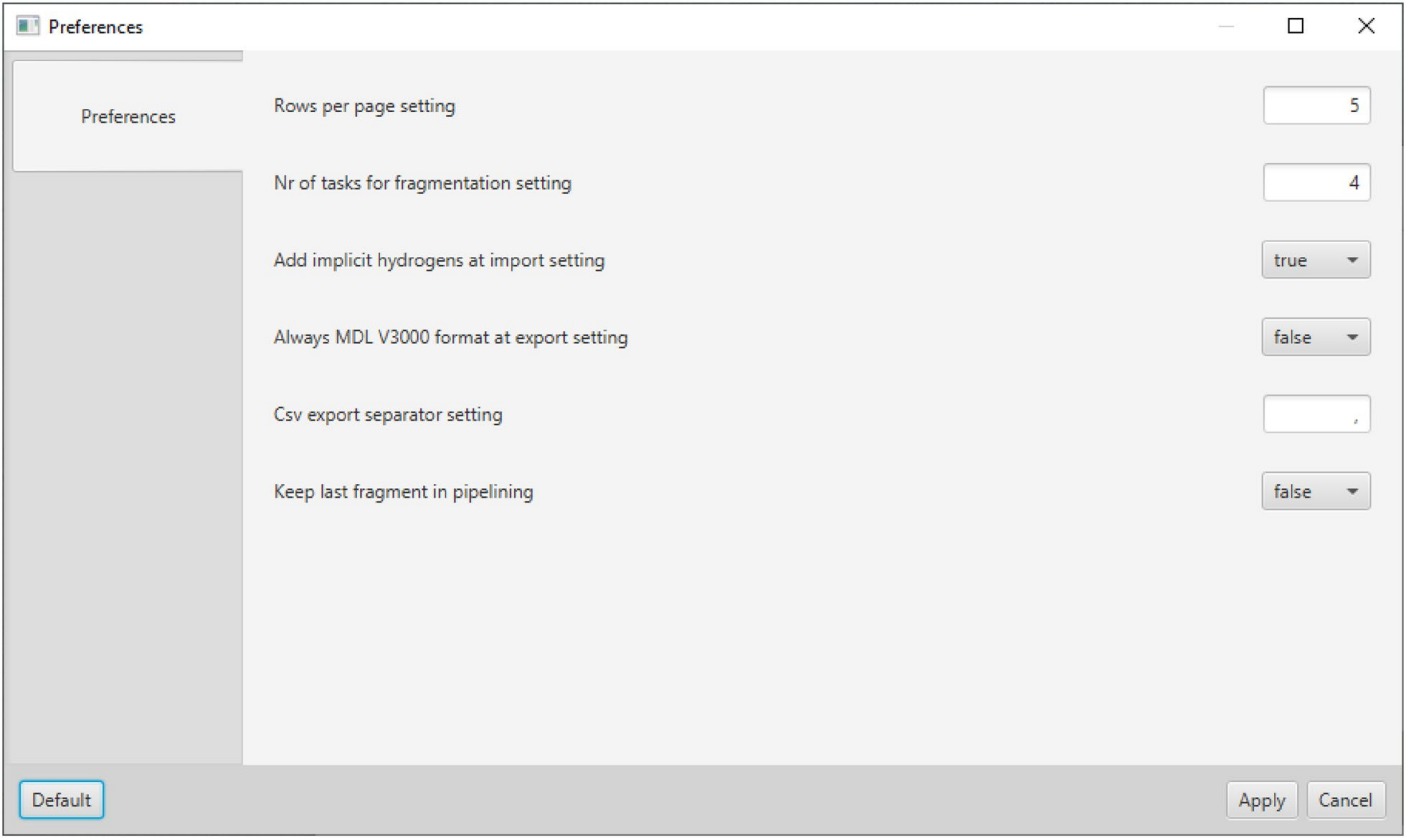
Dialog for general MORTAR preferences. In addition to settings for the GUI, like the number of molecules per page, settings can also be made for importing sets of molecules and result export

The settings of the individual fragmentation algorithms and the MORTAR application in general can be adjusted via separate dialogs (see Figs. 4 and 2). The view for the settings of the algorithms is automatically extended for a new algorithm when it is integrated. All settings are made persistent by line-based text files.

### Fragmentation results

Two new tabs open when a fragmentation job is completed: *Fragments* tab and *Items* tab. If multiple jobs are executed consecutively, the two result tabs are generated for each of them and remain open. The content of result views can be exported as a PDF file or as a text file in CSV format. The PDF file contains the 2D structures in addition to the information displayed as text. The resulting fragments can be exported as SD, Mol, or PDB files. The librepdf library version 1.3.26 [46] is used for PDF export.

### Fragments tab

**Fig. 5:**
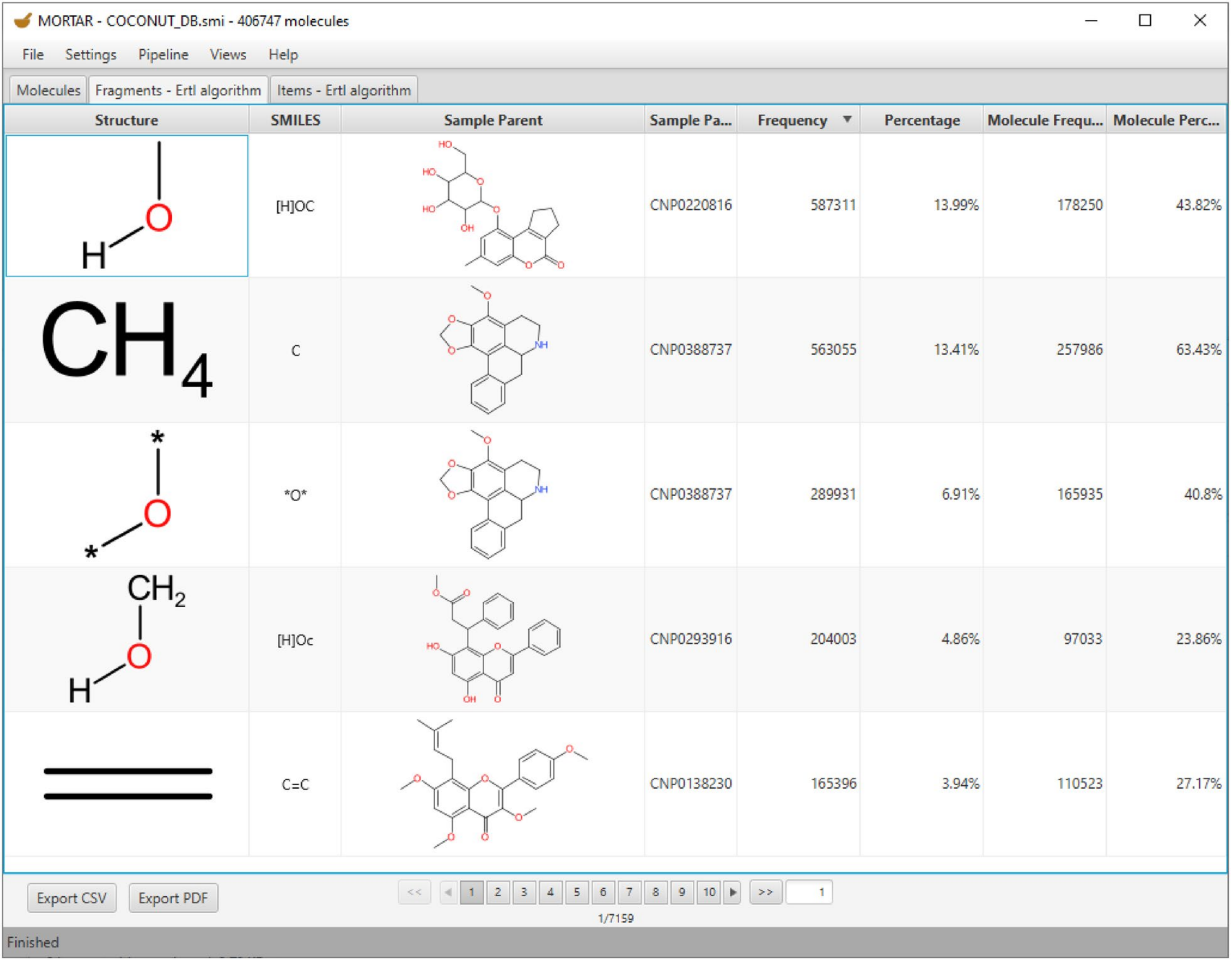
*Fragments* tab with the generated functional group and alkane fragments of the COCONUT database sorted in descending order of frequency

The *Fragments* tab visualises the resulting fragments. Similar to the *Molecules* tab, the resulting fragments are displayed on multiple pages. In addition to the SMILES representation and the 2D structure of each fragment, one column shows a randomly selected parent molecule of the corresponding fragment (see Fig. 5). The *Frequency* column indicates how often the corresponding fragment appears in the fragmented set of molecules. The column *Molecule Frequency* contains the number of molecules in which this fragment appears. In Fig. 5, the five most frequent fragments (functional groups and alkane remnants) resulting from the Ertl algorithm analysis of the COCONUT natural product database are displayed. The most frequent fragment is a hydroxy group connected to an aliphatic carbon atom. Almost as frequently, a single aliphatic carbon atom or methyl group was detected, followed by an ether group that is only half as frequent. In fourth place, there is a hydroxy group connected to an aromatic carbon atom, as it results, i.a., from a phenol (the aromatic character of the attached carbon atom is indicated in the SMILES representation of the group, “[H]Oc”, by lower-case letter notation; this corresponds to the fragment depiction where the carbon atom is not fully saturated with hydrogen atoms). The fifth most frequent FG is an alkene group occurring 165,396 times. The fragments are sorted according to their absolute frequency in Fig. 5. If they would be sorted according to their molecule frequency, the ranking would be different with the methyl group being at the top. The comparatively high number of hydroxy groups detected in absolute numbers as opposed to the molecule frequency means that natural products usually have multiple substituents of this type (3.3 on average, dividing the frequency by the molecule frequency). One type of structure that may be responsible for this trend is glycosidic moieties that occur frequently in natural products [30]. The results of this proof-of-concept analysis using MORTAR are in general agreement with an analogous systematic study of functional group frequencies in natural products conducted by Ertl et al. [28].

### *Items* tab

**Fig. 6:**
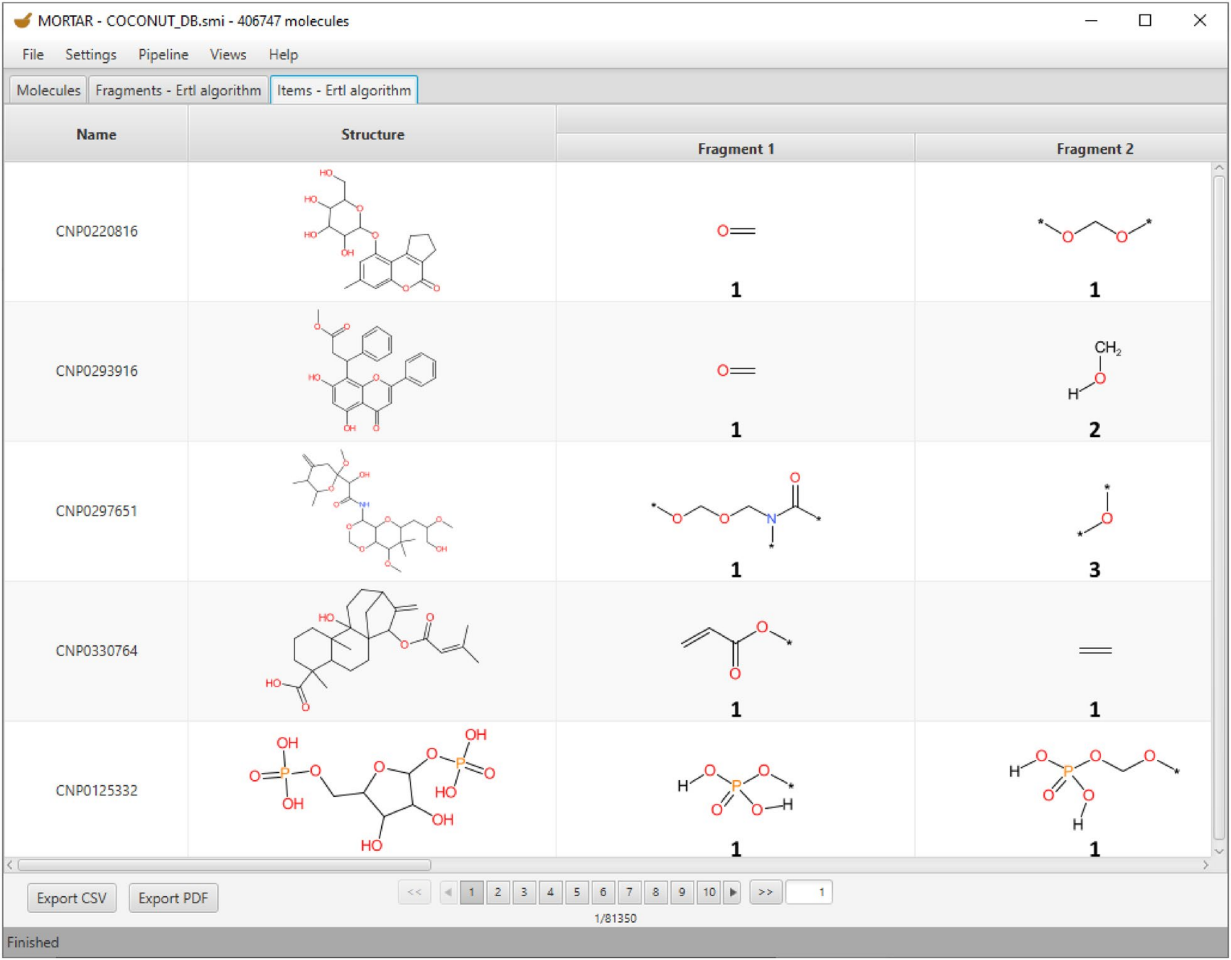
*Items* tab with five molecules from the COCONUT database fragmented with ErtlFunctionalGroupsFinder and the corresponding fragments including their frequencies in the molecule

The *Items* tab also visualises the results of a fragmentation process. Here, however, the fragments are assigned to their individual molecules from the originally imported set. Each molecule is shown with its name, its 2D structure, and the 2D structures of its fragments, including the frequencies of how often the respective fragment appears in this molecule (see Fig. 6).

### Pipelining

**Fig. 7:**
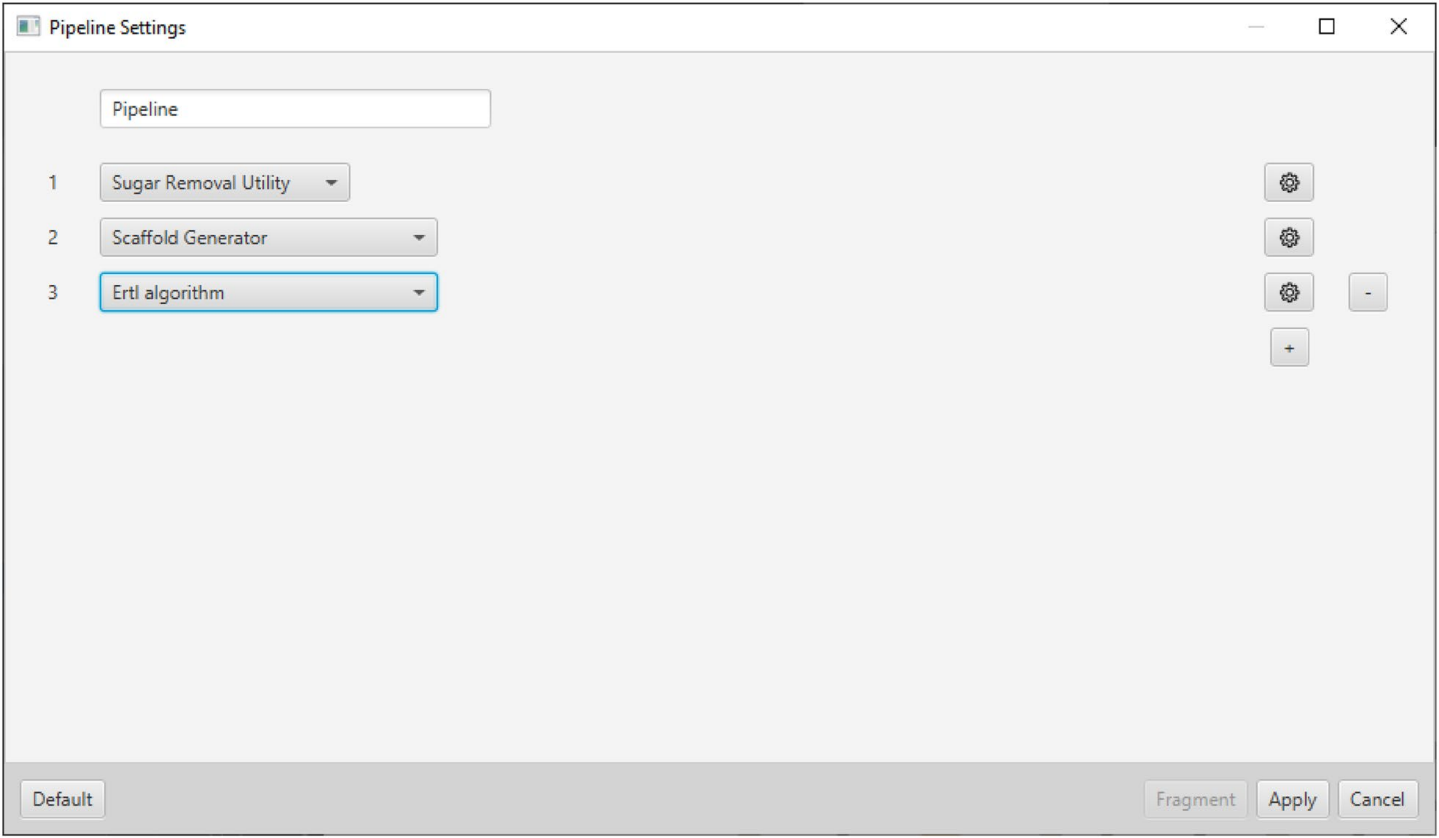
Pipeline settings view shows a pipeline named “Pipeline” with three methods: first, Sugar Removal Utility, second, Scaffold Generator, and the ErtlFunctionalGroupsFinder (Ertl algorithm) as the final step. The gear buttons on the right can be used to adjust the settings for each algorithm. The plus adds another algorithm step and the minus removes the last one. Fragmentation can be started via the *Fragment* button

In addition to executing a single method of fragmentation, MORTAR offers the option of executing fragmentation pipelines, which can be defined and executed with any combination of the integrated fragmentation algorithms where each selected algorithm may have its own individual settings. The *Pipeline Settings* view (see Fig. 7) provides a straightforward way to create a pipeline by adding new methods via buttons and choice boxes. A simple application example of the pipelining functionality is to apply a Sugar Removal Utility (SRU) processing step to remove terminal glycosidic moieties from the studied molecules, as it is usually done in chemical space analyses prior to another fragmentation step to avoid redundancies.

**Fig. 8:**
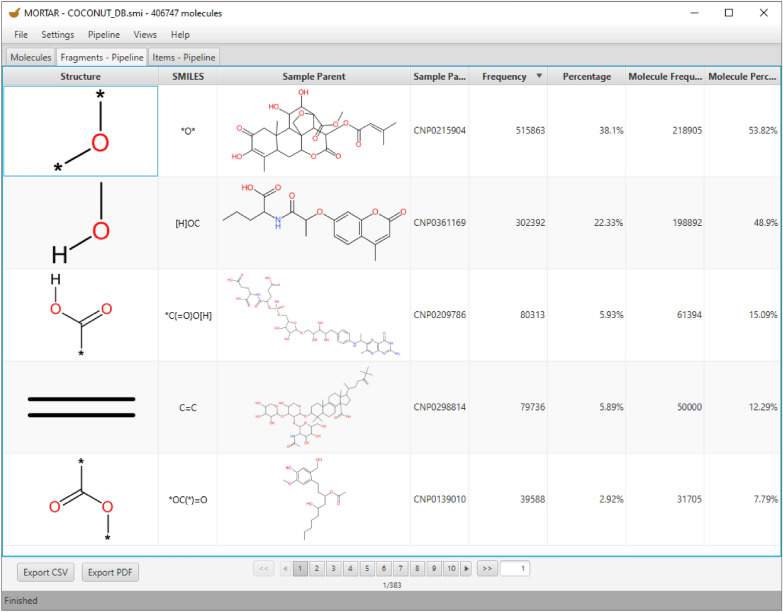
*Fragments* tab showing the results of the pipeline fragmentation approach from above (see Fig. 7). The results of a pipeline approach are presented in the same way as a fragmentation with a single algorithm

A more sophisticated example study for the MORTAR pipelining functionality is inspired by the recent work of Peter Ertl to identify the most common substituents in natural products [47]. Here, NP structures were first deglycosylated, and (ring) substituents were recursively extracted. To set up a similar pipeline in MORTAR, the first step has to be a Sugar Removal Utility processing configured to only return aglycones of input structures. The second step would be a Scaffold Generator fragmentation that only returns side chains. A recursive fragmentation of the side chains is currently not included in MORTAR but for demonstration purposes, an Ertl algorithm processing can be chosen to extract functional groups from them. This pipeline is shown in Fig. 7 and described in more detail in the MORTAR tutorial that can be found on GitHub [48]. Figure 8 depicts the five most frequent functional groups resulting when this pipeline is applied to the natural product structures taken from COCONUT. The most frequent functional group identified in ring side chains is an ether or a hydroxy group (both belong to the same group because the bonds to ring atoms are cut without preserving any information). Following is a hydroxy group connected to an aliphatic carbon atom. In the depicted *Sample Parent* structure in row 2, this group results from the ether group that connects the side chain with the ring. When it is cleaved and saturated with hydrogen, a hydroxy group results. The third functional group is a carboxylic acid functionality. The alkene functionality is identified as the fourth-frequent substituent, followed by the ester functionality.

### Histogram view

**Fig. 9:**
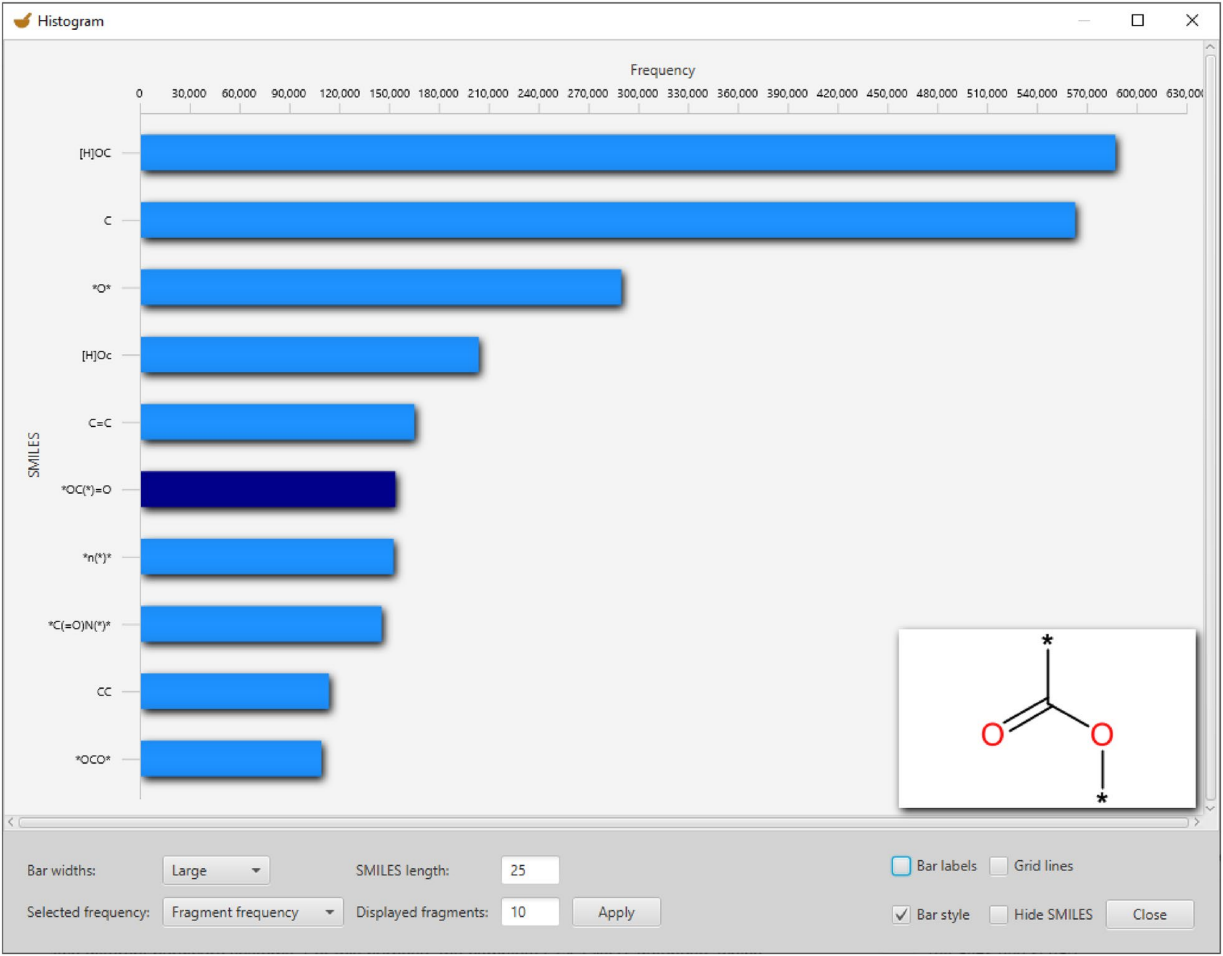
Histogram of the ten most frequent functional groups and alkane remnants found in COCONUT by ErtlFunctionalGroupsFinder. The absolute frequency of the fragments in the resulting fragments set is displayed

To get an overview of the fragment frequencies, a histogram can be created with both types of frequency—the Frequency, which indicates how often the respective fragment occurs in the fragmented set of molecules, or the Molecule Frequency, which indicates the number of molecules in which a fragment occurs. Figure 9 shows a MORTAR histogram view of the ten most frequent fragments of the COCONUT database fragmented with ErtlFunctionalGroupsFinder in default settings (compare Fig. 5). The fragments are sorted according to their absolute frequencies and can also be displayed as a 2D structure image by hovering over the bar of the desired fragment. The fragment structure image is displayed in the lower right corner of the histogram.

### Performance

Performance snapshots of MORTAR were performed for different fragmentation processes on two different hardware systems. For this purpose, the complete COCONUT database was used first. The fragmentation algorithm employed for the first snapshot was ErtlFunctionalGroupsFinder with default settings. On a standard notebook using eight cores of an Intel(R) Core(TM) i7-8750H CPU [49] and allocating 20 GB of memory to the JVM, MORTAR took 100 s to decompose the 406,747 molecules of the COCONUT database imported as SMILES codes into 35,791 fragments and post-process them. On the same machine with the same configuration, MORTAR needed 154 s to decompose the COCONUT database using the Sugar Removal Utility with default settings. Using the Scaffold Generator (*Fragmentation type setting* set to *SCAFFOLD_ONLY*, i.e., generate the molecular scaffold of a structure but do not dissect it further; default settings for the rest) with eight cores on the machine described above, MORTAR took 64 s to process COCONUT.

Using 12 cores of an Intel(R) Xeon(R) Gold Processor 6254 workstation CPU [50] and 250 GB memory for the JVM resulted in a computation time of 229 s to fragment the 2,136,187 molecules of the ChEMBL30 database [51, 52] into 62,722 distinct fragments using ErtlFunctionalGroupsFinder with default settings. With more than 24 parallelised threads, no further performance increase could be achieved.

## Conclusion

This work presents MORTAR, an end-user GUI application for in silico fragmentation and substructure analysis that does not require programming skills. An insight into the chemical space of the substructures of a set of molecules can be obtained with multiple result visualisation options. The pipeline approach makes it possible to work with multiple, sequentially applied methods on one data set. As a Java application, MORTAR runs on all major platforms (Windows, Linux, and macOS). An installer executable is provided especially for Windows. Through the *IMoleculeFragmenter* Java interface class, MORTAR offers a straightforward way to integrate new fragmentation algorithms to support development purposes. Example studies with MORTAR include the investigation of the natural product chemical space by which functional groups frequently appear in their structures, together with a comparison to non-natural product compounds [28], and analogous studies on structures relevant to medicinal chemistry [53]. Even more sophisticated analyses like the investigation of natural product substituents [47] can be attempted in a similar fashion using MORTAR’s pipelining functionality. Looking at specific substructures and their frequencies in a structural database can also be used to access its chemical diversity. This dates back to Bemis and Murcko’s investigation of the diversity of drug molecules known in their time by looking at how many different molecular frameworks can be identified and which of them appear very frequently [21]. A specific application of MORTAR could be the automated generation of adequate fragment molecule sets for mesoscopic simulation approaches of large molecular ensembles to be studied on the nanometer length and microsecond time scales (like “bottom-up” Dissipative Particle Dynamics (DPD) [54] supported by the MFsim/Jdpd [55, 56] environment).

## Availability and requirements

- Project name: MORTAR
- Project home page: https://github.com/FelixBaensch/MORTAR
- Current version: V1.0.5.0
- DOI of archived current version: https://doi.org/10.5281/zenodo.7194013
- Operating system(s): Windows (× 64), macOS (× 64 and AArch64), Linux (× 64 and AArch64)
- Programming language: Java
- Other requirements: Java v17.0.4 or higher, Gradle v7.3 or higher
- Licence: GPL-3.0 Licence
- Any restrictions to use by non-academics: None

## Data Availability

Data and software are freely available under the GPL-3.0 Licence. The source code of MORTAR is available on GitHub at https://github.com/FelixBaensch/MORTAR.

## Abbreviations

2D: Two-dimensional
API: Application Programming Interface
CASE: Computer-Assisted Structure Elucidation
CDK: Chemistry Development Kit
COCONUT: COlleCtion of Open Natural prodUcTs
CPU: Central Processing Unit
CSV: Comma-Separated Value
DPD: Dissipative Particle Dynamics
FG: Functional Group(s)
GUI: Graphical User Interface
HOSE: Hierarchically Ordered Spherical description of Environment
JVM: Java Virtual Machine
MORTAR: MOlecule fRagmenTAtion fRamework
MVC: Model-View-Controller
NP: Natural Product(s)
OS: Operating System
PDB: Protein Data Bank
PDF: Portable Document Format
R: Registered trademark
RAM: Random-Access Memory
SD(F): Structure Data (File)
SMILES: Simplified Molecular Line Entry System
SRU: Sugar Removal Utility
TM: Unregistered TradeMark
